# 132. Standardized Antimicrobial Administration Ratios to Guide Antimicrobial Stewardship in the Neonatal Intensive Care Unit: a Single Center Experience

**DOI:** 10.1093/ofid/ofab466.334

**Published:** 2021-12-04

**Authors:** Steven Smoke, Uzma Hasan, Eileen Steffen, Kamtorn Vangvanichyakorn

**Affiliations:** Saint Barnabas Medical Center, Milltown, New Jersey

## Abstract

**Background:**

Antimicrobial stewardship (AMS) is particularly challenging in the neonatal population. Both under- and overuse can negatively impact outcomes. There are limited reports of strategies to improve AMS in the neonatal population. Standardized Antimicrobial Administration Ratios (SAARs) are novel metrics of antimicrobial use, recently introduced for neonatal populations by the National Healthcare Safety Network (NHSN). We describe our experience using SAARs to guide AMS in the neonatal intensive care unit (NICU).

**Methods:**

This was a retrospective study conducted from January 2020 to April 2021. A team consisting of AMS and NICU department staff identified and implemented AMS strategies. Based on a review of NICU SAAR data, a goal was set to reduce third generation cephalosporin use by encouraging aminoglycoside use when appropriate. The pre-implementation period was January 2020 to May 2020 and the post-implementation period was July 2020 to April 2021. Antibiotic use was measured as SAARs and compared between study periods. The primary outcome was the neonatal SAAR for third generation cephalosporins. Secondary outcomes included SAARs for aminoglycosides and all neonatal antibacterial agents. SAARs were compared using the NHSN Statistics Calculator.

**Results:**

For third generation cephalosporins, there were 385 observed antimicrobial days (OAD) and 115 expected antimicrobial days (EAD) in the pre-implementation period compared to 597 OAD and 228 EAD in the post implementation period. This resulted in a SAAR of 3.34 and 2.62, respectively; a reduction of 22% (p < 0.001). For aminoglycosides, there were 713 OAD and 584 EAD compared to 1617 OAD and 1155 EAD. This resulted in a SAAR of 1.22 and 1.4; an increase of 15% (p = 0.002). For all neonatal antibacterial agents, there were 2716 OAD and 1739 EAD compared to 5321 OAD and 3438 EAD. This resulted in a SAAR of 1.56 and 1.55; indicating no change in use (p = 0.70). See Table 1 for results.

Table 1. Antibiotic Use

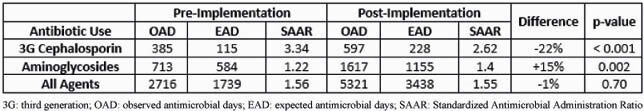

**Conclusion:**

While this initiative resulted in decreased use of third generation cephalosporins, this was not associated with a decrease in antibiotic use overall. Use of SAARs in the NICU may be helpful in both identifying opportunities to improve antibiotic use and monitoring antibiotic use over time.

**Disclosures:**

**Steven Smoke, PharmD, Karius **(Advisor or Review Panel member) **Shionogi** (Scientific Research Study Investigator, Advisor or Review Panel member)

